# Unusual location of sinonasal ossifying fibroma of the posterior ethmoid sinus: a case report and literature review

**DOI:** 10.1097/RC9.0000000000000915

**Published:** 2026-09-09

**Authors:** Abdulrahman S. Altowaim, Yazieed Mohammed AlBarrak, Lenah Khalid Al Fadhil, Abdulaziz Mohammed Alghamdi

**Affiliations:** a College of Medicine, King Saud Bin Abdulaziz University for Health Sciences, Riyadh, Saudi Arabia; b Department of Otolaryngology–Head and Neck Surgery, Prince Sultan Military Medical City, Riyadh, Saudi Arabia; c Department of Otolaryngology–Head and Neck Surgery, King Fahad Military Medical Complex, Dhahran, Saudi Arabia

**Keywords:** benign fibro-osseous lesion, case report, endoscopic sinus surgery, ethmoid sinus, ossifying fibroma, skull base reconstruction

## Abstract

**Introduction and importance:**

Ossifying fibroma is a benign, well-demarcated fibro-osseous neoplasm that most commonly affects the mandible and maxilla. Involvement of the paranasal sinuses is rare, and ethmoid sinus localization is particularly uncommon. Because sinonasal ossifying fibroma may mimic other fibro-osseous and osseous lesions radiologically, histopathologic confirmation and complete excision are important for diagnosis and disease control.

**Case presentation:**

A 43-year-old Saudi man with well-controlled type 2 diabetes mellitus presented with a 6-month history of progressive right-sided nasal obstruction and frontal headache. He reported no history of epistaxis, visual disturbance, or anosmia. Computed tomography demonstrated a well-defined 2.5 × 1.6 × 2.3 cm sclerotic lesion with ground-glass matrix occupying the right posterior ethmoid air cells and abutting the skull base. Histopathology confirmed ossifying fibroma. The patient underwent complete endoscopic transnasal resection with skull base repair using a free autologous cartilage graft. The clinical course is summarized in Table 1. Recovery was uneventful, and he was discharged on postoperative day two.

**Clinical discussion:**

Ethmoidal ossifying fibroma is rare and may present with nonspecific sinonasal symptoms. Complete excision is recommended for symptomatic sinonasal disease because incomplete removal is associated with recurrence. In selected cases, an endoscopic approach can provide direct visualization, avoid external approach-related morbidity, and allow skull base reconstruction when required. However, 4-month follow-up is insufficient to make definitive statements about long-term recurrence.

**Conclusion:**

Posterior ethmoid ossifying fibroma is an uncommon presentation of a benign fibro-osseous tumor. Accurate diagnosis requires clinical, radiologic, and histopathologic correlation. Complete endoscopic resection with appropriate skull base repair can achieve favorable short-term outcomes in selected cases, but prolonged surveillance is necessary.

## Introduction

Ossifying fibroma (OF) is a benign fibro-osseous neoplasm characterized by replacement of normal bone with cellular fibrous tissue containing variably mineralized material. It most frequently arises in the jaw bones, particularly the mandible, whereas involvement of the paranasal sinuses is rare. When OF occurs in the sinonasal tract, it may involve the maxillary, frontal, ethmoid, or sphenoid sinuses and may extend toward the orbit or anterior skull base depending on its location and size^[^1–6^]^.

HIGHLIGHTS

- Ossifying fibroma rarely involves the paranasal sinuses, and posterior ethmoid sinus involvement is particularly uncommon.
- Radiologic-histopathologic correlation is essential because sinonasal fibro-osseous lesions have overlapping appearances.
- Complete endoscopic transnasal resection can avoid external-incision morbidity in carefully selected ethmoid lesions.
- Skull base reconstruction may be required when ethmoid disease abuts or violates the skull base.
- Short-term symptom resolution is encouraging, but long-term surveillance remains necessary because recurrence can occur late.

The rarity of paranasal sinus OF is clinically relevant. A systematic review of nose and paranasal sinus OF identified 137 patients in 103 reports, but recurrence data were limited by inconsistent follow-up^[^1^]^. Sinonasal case series have similarly emphasized that these tumors are uncommon, locally expansile, and diagnostically challenging^[^2–4^]^. Ethmoid sinus involvement is especially unusual and important because this region is adjacent to the lamina papyracea, orbit, anterior ethmoid artery, cribriform plate, and anterior skull base. Even histologically benign disease in this location may therefore create surgical and reconstructive challenges^[^7–13^]^.

Symptoms depend on the site and extent of disease and may include nasal obstruction, headache, facial pressure, anosmia, epistaxis, proptosis, diplopia, visual disturbance, or recurrent sinusitis; some lesions are incidentally discovered^[^1,2,9–13^]^. The differential diagnosis includes fibrous dysplasia, osteoma, osteoblastoma, aneurysmal bone cyst, juvenile trabecular OF, juvenile psammomatoid OF, and malignant bone-forming tumors. Radiologic demarcation, internal mineralization, growth pattern, and histopathologic architecture help distinguish these entities, although overlap is common^[^5–8,14,15^]^.

Complete resection is generally recommended for symptomatic sinonasal OF or lesions involving critical areas, because incomplete excision has been linked to recurrence^[^1–4,8,16–18^]^. Modern endoscopic endonasal techniques have expanded the role of minimally invasive surgery for selected paranasal sinus and skull base-adjacent lesions by improving visualization and reducing external-incision morbidity^[^3,9,11,18–20^]^. This case report has been prepared in accordance with the SCARE 2025 guidelines ^[^21^]^.

## Case presentation

A 43-year-old Saudi man with well-controlled type 2 diabetes mellitus presented to the otolaryngology clinic with a 6-month history of progressive right-sided nasal obstruction and a persistent frontal headache. He reported no history of epistaxis, visual disturbances, anosmia, previous sinonasal surgery, trauma, or a family history of bone disease. He was a non-smoker.

Anterior rhinoscopy demonstrated a high septal deviation and bilateral inferior turbinate hypertrophy, with narrowing of the right nasal cavity. No obvious intranasal mass was visible on anterior examination. Neurologic and ophthalmologic screening examinations were unremarkable.

Computed tomography of the paranasal sinuses showed a well-defined 2.5 × 1.6 × 2.3 cm sclerotic lesion with a ground-glass matrix occupying the right posterior ethmoid air cells and abutting the skull base, with local mass effect but no radiologic features of frank malignancy (Fig. 1). The leading differential diagnoses included OF, fibrous dysplasia, osteoma, and other benign fibro-osseous lesions. Endoscopic biopsy was performed. Histopathologic examination demonstrated separate bone fragments with variably stellate fibrous stroma and mineralized material, including cementum-like basophilic deposits and woven bone rimmed by osteoblasts, confirming OF (Fig. 2).

**Figure 1.**
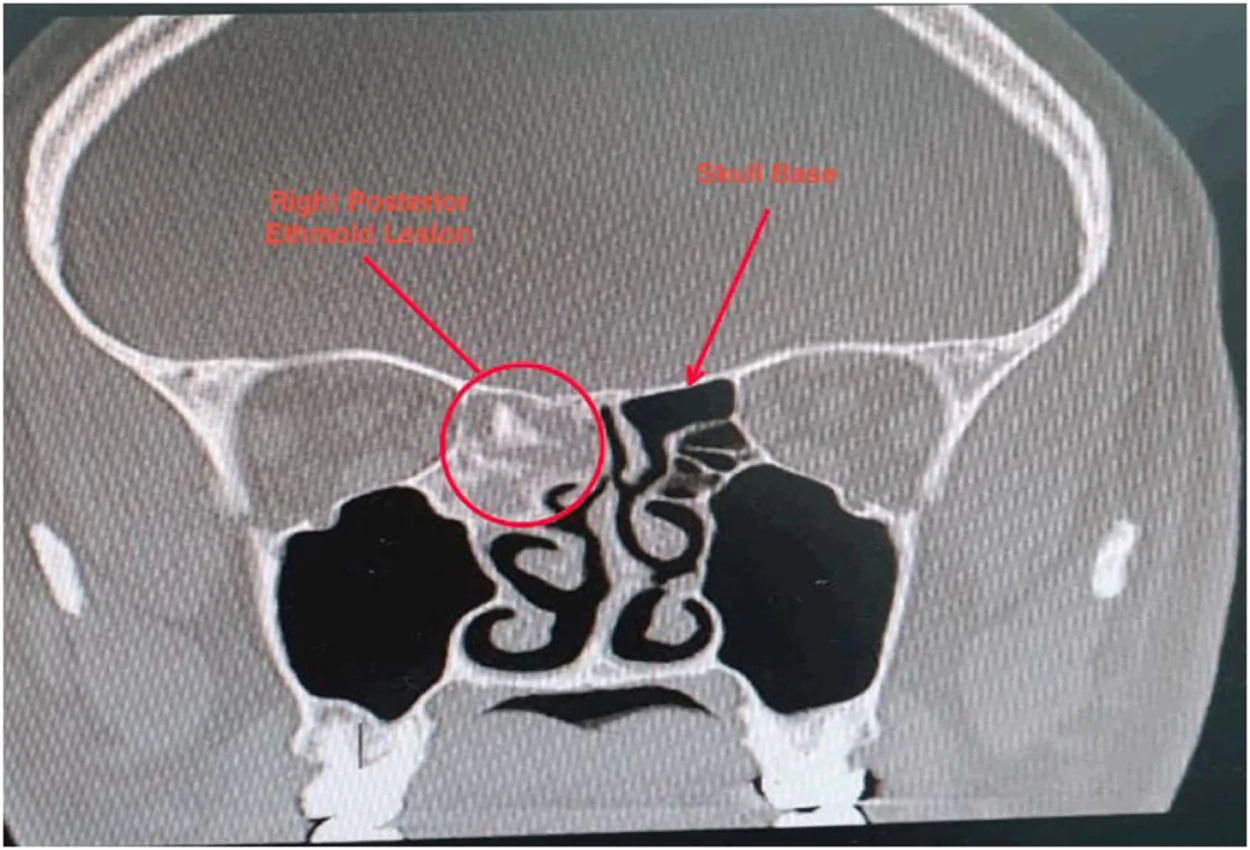
Annotated coronal computed tomography (CT) scan of the paranasal sinuses showing a well-defined sclerotic lesion with a ground-glass matrix occupying the right posterior ethmoid air cells and abutting the skull base. The available CT image has been annotated to clarify the lesion site and the adjacent critical anatomy.

**Figure 2.**
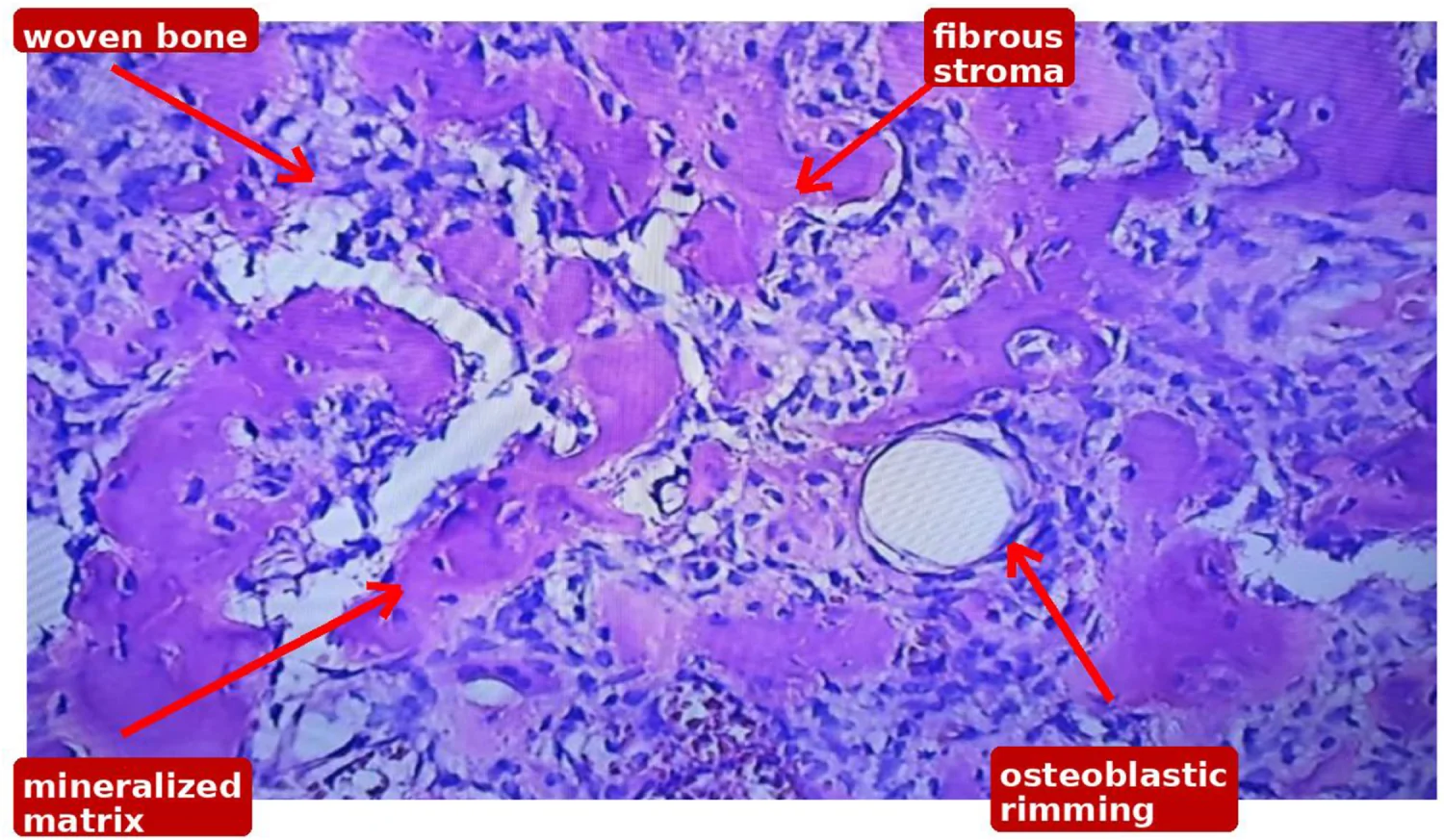
Annotated histopathologic examination of the ethmoidal lesion, showing fibro-osseous tissue composed of cellular fibrous stroma with variable mineralization, woven bone, osteoblastic rimming, and cementum-like basophilic and mineralized deposits, consistent with ossifying fibroma (hematoxylin and eosin stain).

After optimization of glycemic control, the patient underwent endoscopic transnasal resection under general anesthesia. Complete resection was achieved through a transnasal endoscopic approach using standard endoscopic sinus instrumentation. The operation was performed without an external incision. A focal skull base defect was identified intraoperatively after lesion removal and was repaired using a multilayer technique centered on a free autologous cartilage graft (Fig. 3). No intraoperative or immediate postoperative complications were recorded, and blood loss was minimal.

**Figure 3.**
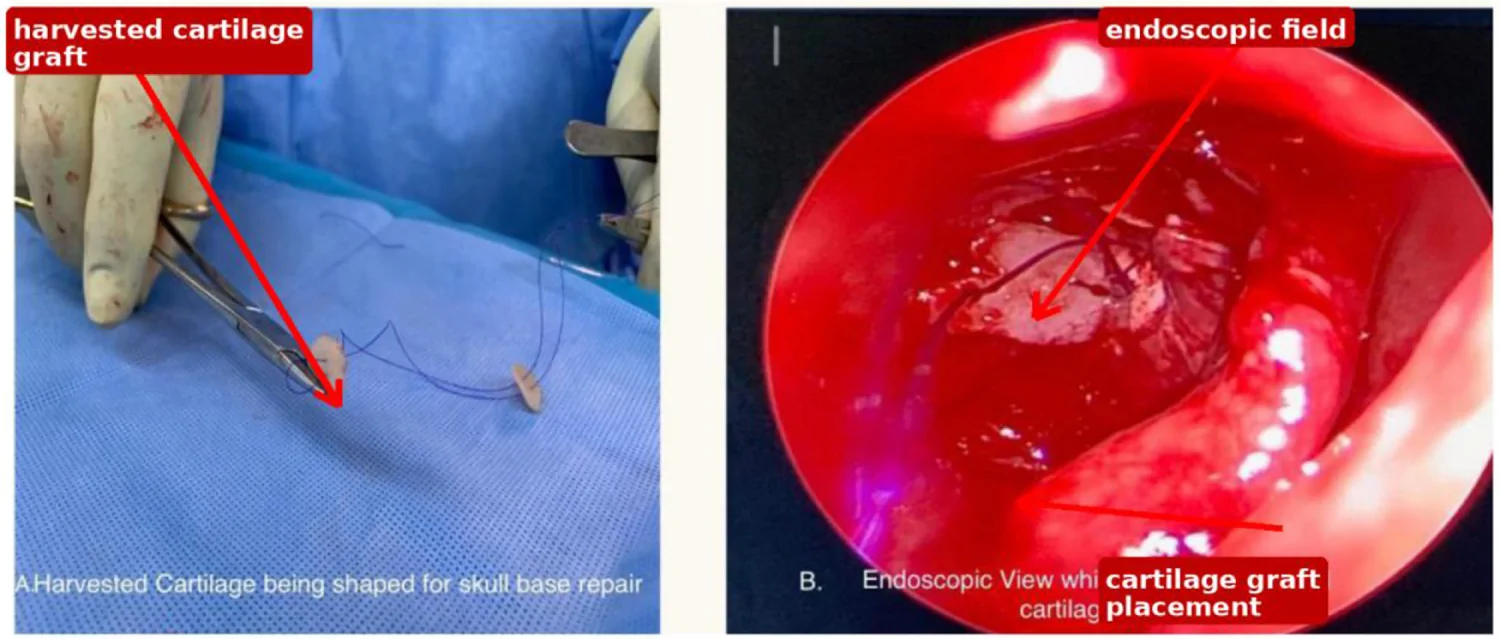
Annotated intraoperative images showing the preparation of a harvested cartilage graft and its endoscopic placement during multilayer skull base reconstruction after complete resection of the posterior ethmoid ossifying fibroma. The available intraoperative image set demonstrates the reconstruction rather than the entire resection sequence.

The patient was discharged on postoperative day two with routine postoperative nasal care instructions and outpatient follow-up. At the 2-week follow-up, the sinonasal cavity was healing appropriately. At the 4-month follow-up, the patient reported complete resolution of nasal obstruction and headache, with no clinical evidence of complications or recurrence. The clinical timeline is presented in Table 1. Surveillance computed tomography is planned at 1 year, with continued long-term endoscopic follow-up.

**Table 1:** Clinical timeline of presentation, diagnosis, endoscopic management, follow-up, and planned surveillance.

Time point	Clinical event
Six-months before presentation	Progressive right-sided nasal obstruction and frontal headache developed.
Initial clinic evaluation	Anterior rhinoscopy showed high septal deviation, bilateral inferior turbinate hypertrophy, and right nasal cavity narrowing without an obvious anterior intranasal mass. Neurologic and ophthalmologic screening examinations were unremarkable.
Diagnostic imaging	Computed tomography of the paranasal sinuses showed a well-defined 2.5 × 1.6 × 2.3 cm sclerotic lesion with ground-glass matrix occupying the right posterior ethmoid air cells and abutting the skull base (Fig. 1).
Endoscopic biopsy	Histopathology demonstrated fibro-osseous tissue with stellate fibrous stroma, cementum-like basophilic deposits, woven bone, and osteoblastic rimming, confirming ossifying fibroma (Fig. 2).
Preoperative preparation	Glycemic control was optimized before definitive surgery.
Definitive surgery	Complete endoscopic transnasal resection was performed under general anesthesia. A focal skull base defect was repaired using a multilayer technique with a free autologous cartilage graft (Fig. 3).
Postoperative day 2	The patient was discharged in stable condition with routine postoperative nasal care instructions.
Two-week follow-up	The sinonasal cavity was healing appropriately without early complications.
Four-month follow-up	The patient reported complete resolution of nasal obstruction and headache, with no clinical evidence of recurrence.
Planned follow-up	Surveillance computed tomography is planned at 1 year with continued long-term endoscopic follow-up.

## Discussion

OF belongs to the broader group of benign fibro-osseous lesions, in which normal bone is replaced by fibrous tissue containing varying amounts of mineralized material^[^5,6^]^. Although the mandible remains the classic site, sinonasal involvement is uncommon and ethmoid sinus disease is particularly rare^[^1–4,9–13,19,22,23^]^. This distinction is important because the behavior and management of fibro-osseous lesions vary by site. Mandibular lesions can often be managed conservatively or with curettage depending on size and symptoms, whereas paranasal sinus lesions are more likely to require complete excision because of anatomic constraints, risk of extension, and recurrence after incomplete removal^[^1–4,16–18^]^.

The present case reinforces several diagnostic principles. First, sinonasal OF often produces nonspecific symptoms. The patient’s obstruction and headache were consistent with mass effect from a posterior ethmoid lesion, but the absence of epistaxis, visual symptoms, and a visible anterior nasal mass could have delayed suspicion of a tumor. Second, radiology alone may not be definitive. Well-demarcated sclerosis and a ground-glass matrix support a benign fibro-osseous process, but overlap with fibrous dysplasia, osteoma, and other osseous lesions remains common^[^6,7,15^]^. Histopathology, therefore, remains essential, especially before definitive surgical planning in skull base-adjacent lesions^[^7^]^.

The literature supports the increasing role of endoscopic surgery in selected sinonasal OFs. Wang *et al* reported 31 endoscopically treated cases and concluded that the endoscopic approach was safe and effective for lesions of the nose and paranasal sinuses^[^3^]^. Appiani *et al* also emphasized that an endoscopic endonasal approach can be appropriate when complete resection is feasible, and the surgeon has adequate access and expertise^[^2^]^. Jurlina *et al* described complete endonasal endoscopic resection of a large ethmoid OF involving the orbit and anterior skull base, illustrating the expanded indications of endoscopic skull base techniques^[^9^]^. Recent reports on ethmoid and skull base-adjacent disease continue to support endoscopic management while emphasizing careful case selection and surveillance^[^11–13,18,20^]^.

Internationally, the main contribution of this case is not merely that the tumor was benign, but that it occurred in a rare posterior ethmoid location adjacent to the skull base and was treated completely through a minimally invasive transnasal route. Compared with earlier external approaches such as transglabellar, subcranial, or craniofacial access^[^23–26^]^, endoscopic resection avoids facial incisions and may reduce morbidity when exposure is adequate. However, external or combined approaches remain necessary for very large, inaccessible, recurrent, or extensively orbital/intracranial lesions^[^2,4,23–25^]^.

Recurrence is a central concern. Published recurrence estimates are difficult to compare because studies mix adult OF, juvenile variants, anatomic sites, surgical approaches, and follow-up durations. The systematic review by Manes *et al* specifically noted that recurrence data were limited by a lack of follow-up^[^1^]^. Juvenile variants, especially psammomatoid lesions, can recur and behave more aggressively^[^8,11,13,14^]^. Therefore, the absence of recurrence at 4 months in the present patient should be interpreted only as a favorable short-term result. Continued nasal endoscopy and interval imaging are necessary, and the planned 1-year CT is appropriate.

## Strengths and limitations

The strengths of this report include the rare posterior ethmoid location, radiologic-histopathologic correlation, complete, minimally invasive management, skull base reconstruction, and an uncomplicated short-term outcome. The case is relevant to rhinologists, skull base surgeons, radiologists, and pathologists because diagnosis and treatment require integration of imaging, endoscopic anatomy, histopathology, and reconstructive planning.

The limitations are also important. First, follow-up is limited to 4 months, which is insufficient to draw strong conclusions regarding recurrence in fibro-osseous lesions. Second, the available imaging set is limited to the CT image included with the submitted material; additional axial and sagittal CT views would better demonstrate lesion extent and its relationship to the orbit and skull base. Third, the intraoperative image set demonstrates cartilage graft preparation and placement but does not fully show lesion exposure, stepwise resection, or the final reconstruction. Fourth, the histopathology image would be stronger with higher-resolution, annotated images. To address these limitations within the available material, the submitted figures have been annotated, the legends expanded, and the need for long-term surveillance explicitly stated.

## Patient perspective

At follow-up, the patient reported complete resolution of nasal obstruction and headache and described substantial improvement in daily comfort after surgery.

## Conclusion

OF rarely arises in the paranasal sinuses, and posterior ethmoidal localization is particularly uncommon. This case highlights the need to consider OF in the differential diagnosis of well-demarcated fibro-osseous ethmoidal lesions, especially when symptoms are nonspecific.

Histopathologic confirmation remains essential because radiologic overlap with other osseous and fibro-osseous lesions is common. Complete endoscopic transnasal resection with skull base repair can be safe and effective in selected cases when performed with appropriate expertise. Long-term endoscopic and radiologic surveillance is necessary because recurrence may occur late, particularly after incomplete excision.

## Data Availability

The data supporting the findings of this case are not publicly available due to institutional and privacy restrictions but are available from the corresponding author upon reasonable request.
